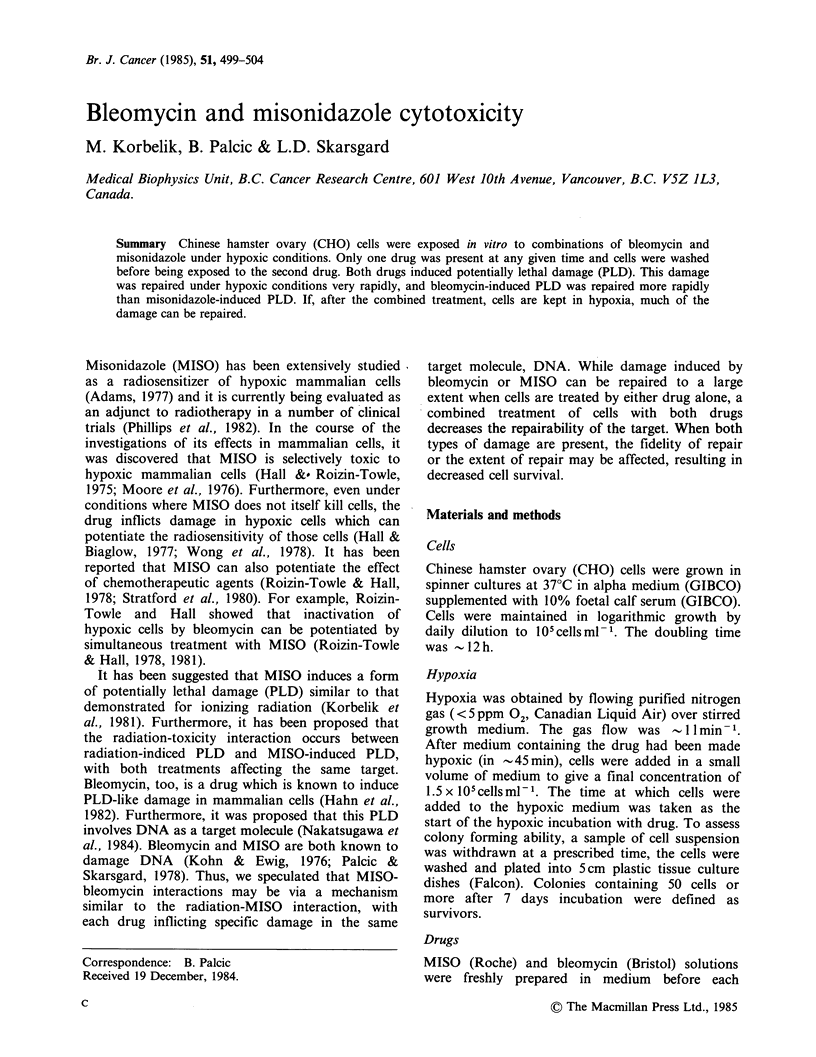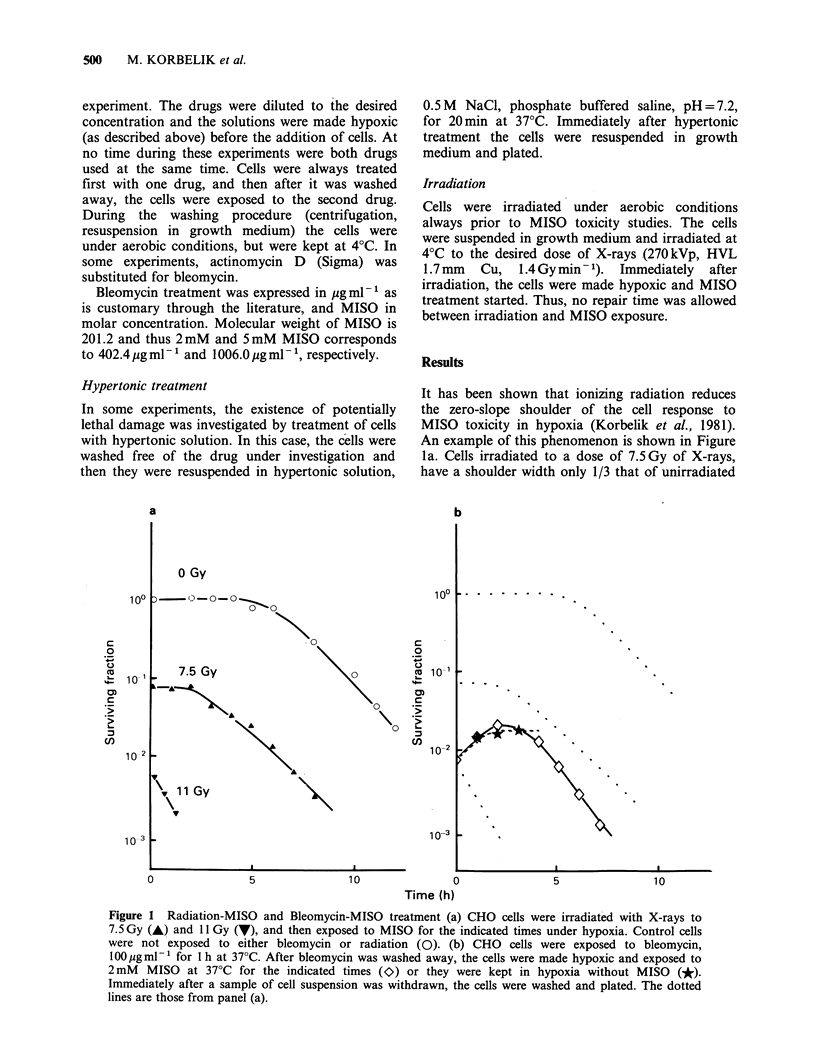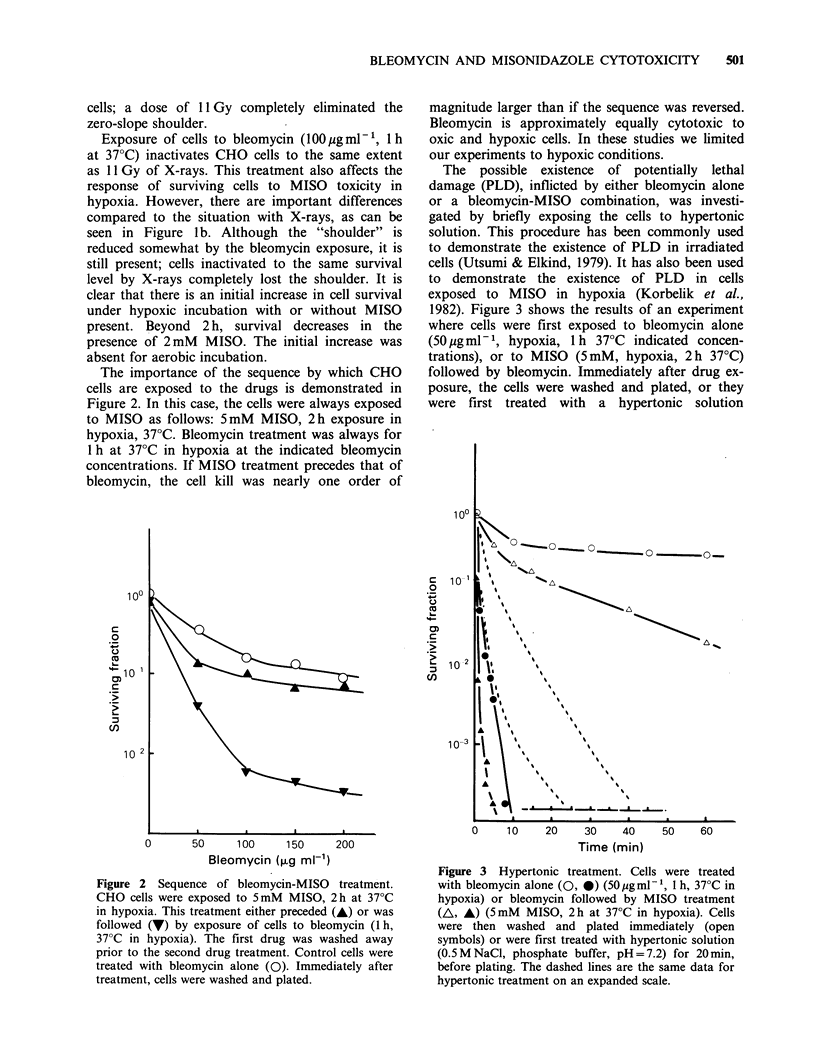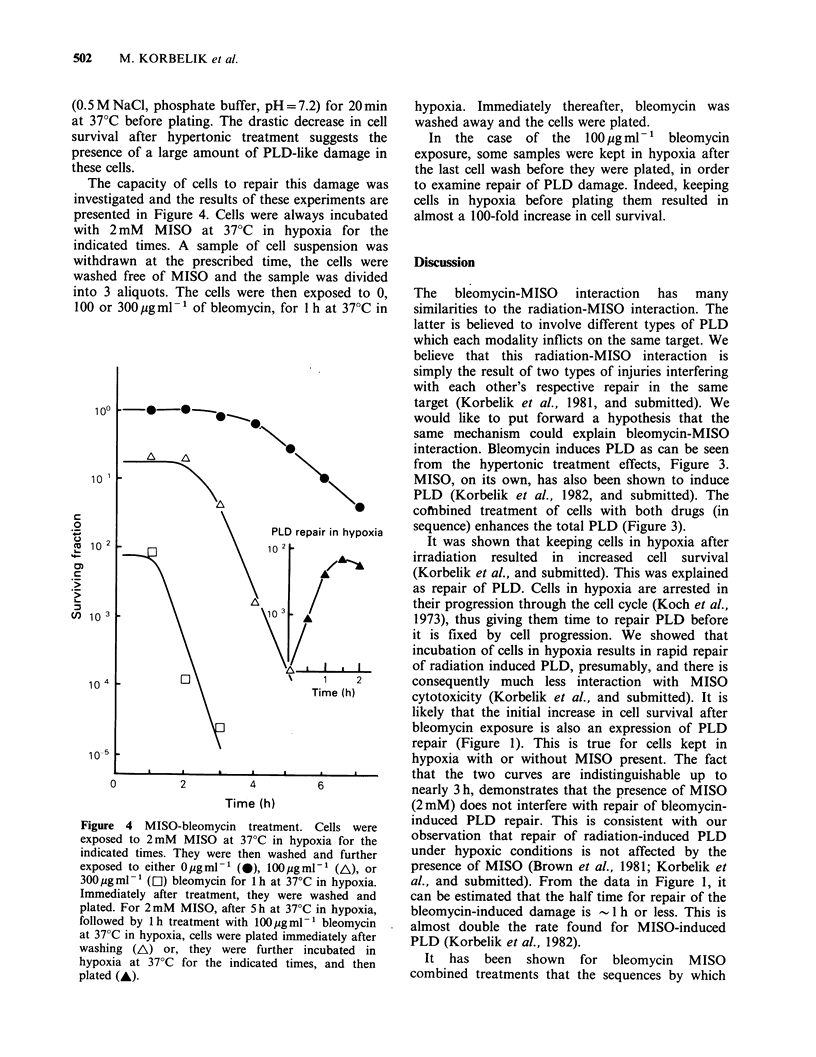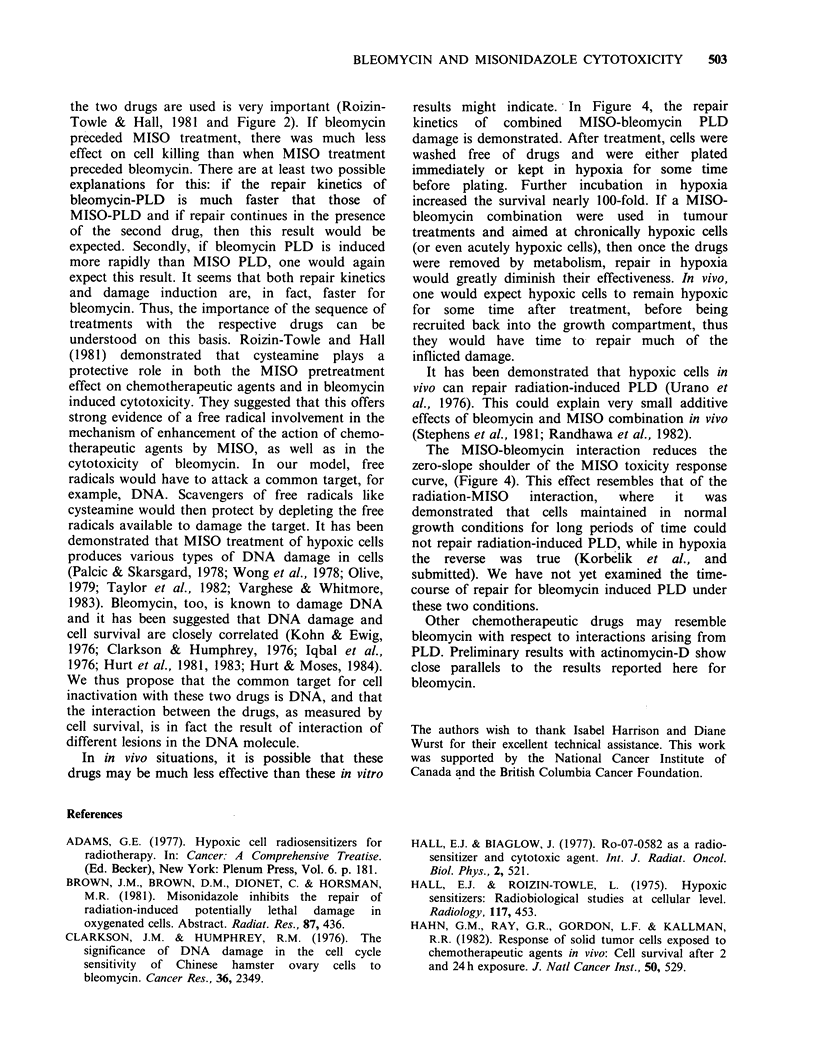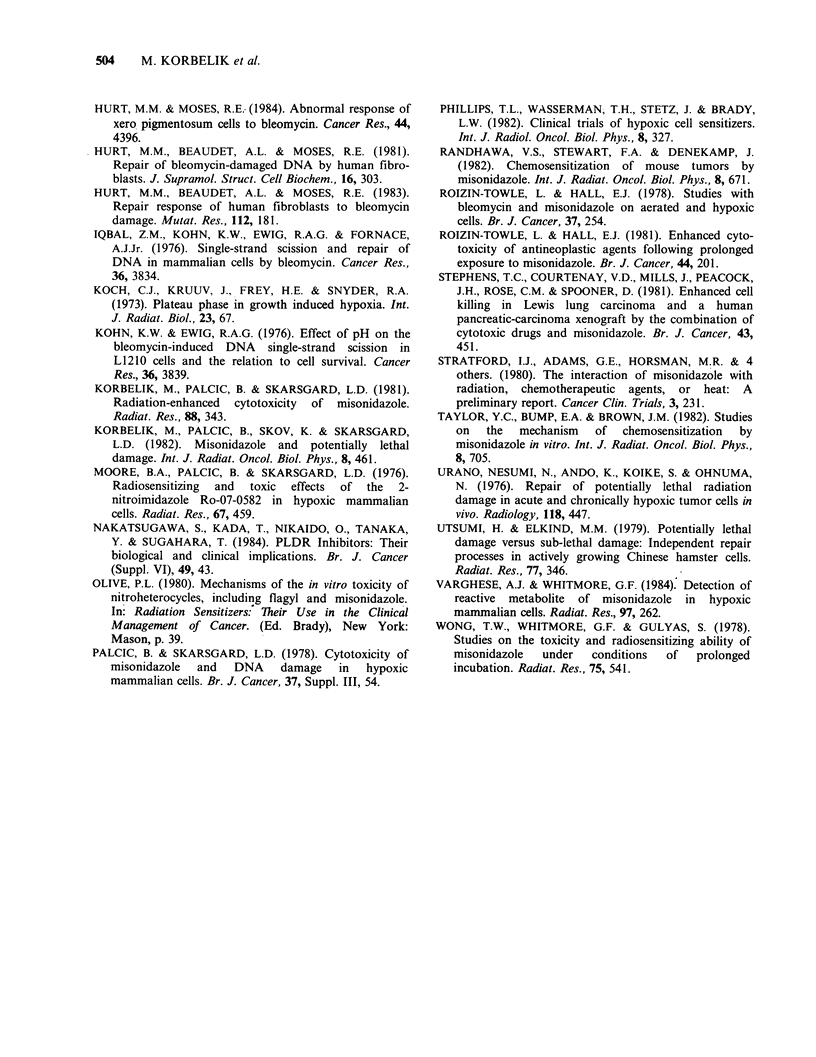# Bleomycin and misonidazole cytotoxicity.

**DOI:** 10.1038/bjc.1985.72

**Published:** 1985-04

**Authors:** M. Korbelik, B. Palcic, L. D. Skarsgard

## Abstract

Chinese hamster ovary (CHO) cells were exposed in vitro to combinations of bleomycin and misonidazole under hypoxic conditions. Only one drug was present at any given time and cells were washed before being exposed to the second drug. Both drugs induced potentially lethal damage (PLD). This damage was repaired under hypoxic conditions very rapidly, and bleomycin-induced PLD was repaired more rapidly than misonidazole-induced PLD. If, after the combined treatment, cells are kept in hypoxia, much of the damage can be repaired.


*Br. J. Cancer* (1985), **51**, 499–504

### Bleomycin and misonidazole cytotoxicity

M. Korbelik, B. Palcic & L.D. Skarsgard

*Medical Biophysics Unit, B.C. Cancer Research Centre, 601 West 10th Avenue, Vancouver, B.C. V5Z 1L3, Canada.*

**Summary** Chinese hamster ovary (CHO) cells were exposed *in vitro* to combinations of bleomycin and misonidazole under hypoxic conditions. Only one drug was present at any given time and cells were washed before being exposed to the second drug. Both drugs induced potentially lethal damage (PLD). This damage was repaired under hypoxic conditions very rapidly, and bleomycin-induced PLD was repaired more rapidly than misonidazole-induced PLD. If, after the combined treatment, cells are kept in hypoxia, much of the damage can be repaired.

Misonidazole (MISO) has been extensively studied as a radiosensitizer of hypoxic mammalian cells (Adams, 1977) and it is currently being evaluated as an adjunct to radiotherapy in a number of clinical trials (Phillips *et al.,* 1982). In the course of the investigations of its effects in mammalian cells, it was discovered that MISO is selectively toxic to hypoxic mammalian cells (Hall & Roizin-Towle, 1975; Moore *et al.,* 1976). Furthermore, even under conditions where MISO does not itself kill cells, the drug inflicts damage in hypoxic cells which can potentiate the radiosensitivity of those cells (Hall & Biaglow, 1977; Wong *et al.,* 1978). It has been reported that MISO can also potentiate the effect of chemotherapeutic agents (Roizin-Towle & Hall, 1978; Stratford *et al.,* 1980). For example, Roizin-Towle and Hall showed that inactivation of hypoxic cells by bleomycin can be potentiated by simultaneous treatment with MISO (Roizin-Towle & Hall, 1978, 1981).

It has been suggested that MISO induces a form of potentially lethal damage (PLD) similar to that demonstrated for ionizing radiation (Korbelik *et al.,* 1981). Furthermore, it has been proposed that the radiation-toxicity interaction occurs between radiation-indiced PLD and MISO-induced PLD, with both treatments affecting the same target. Bleomycin, too, is a drug which is known to induce PLD-like damage in mammalian cells (Hahn *et al.,* 1982). Furthermore, it was proposed that this PLD involves DNA as a target molecule (Nakatsugawa *et al.,* 1984). Bleomycin and MISO are both known to damage DNA (Kohn & Ewig, 1976; Palcic & Skarsgard, 1978). Thus, we speculated that MISO-bleomycin interactions may be via a mechanism similar to the radiation-MISO interaction, with each drug inflicting specific damage in the same target molecule, DNA. While damage induced by bleomycin or MISO can be repaired to a large extent when cells are treated by either drug alone, a combined treatment of cells with both drugs decreases the repairability of the target. When both types of damage are present, the fidelity of repair or the extent of repair may be affected, resulting in decreased cell survival.

### Materials and methods

### *Cells*

Chinese hamster ovary (CHO) cells were grown in spinner cultures at 37°C in alpha medium (GIBCO) supplemented with 10% foetal calf serum (GIBCO). Cells were maintained in logarithmic growth by daily dilution to $10^5$ cells ml$^{-1}$. The doubling time was ~12 h.

### *Hypoxia*

Hypoxia was obtained by flowing purified nitrogen gas (<5 ppm $O_2$, Canadian Liquid Air) over stirred growth medium. The gas flow was ~1 l min$^{-1}$. After medium containing the drug had been made hypoxic (in ~45 min), cells were added in a small volume of medium to give a final concentration of $1.5 \times 10^5$ cells ml$^{-1}$. The time at which cells were added to the hypoxic medium was taken as the start of the hypoxic incubation with drug. To assess colony forming ability, a sample of cell suspension was withdrawn at a prescribed time, the cells were washed and plated into 5 cm plastic tissue culture dishes (Falcon). Colonies containing 50 cells or more after 7 days incubation were defined as survivors.

### *Drugs*

MISO (Roche) and bleomycin (Bristol) solutions were freshly prepared in medium before each

Correspondence: B. Palcic
Received 19 December, 1984.

© The Macmillan Press Ltd., 1985

experiment. The drugs were diluted to the desired concentration and the solutions were made hypoxic (as described above) before the addition of cells. At no time during these experiments were both drugs used at the same time. Cells were always treated first with one drug, and then after it was washed away, the cells were exposed to the second drug. During the washing procedure (centrifugation, resuspension in growth medium) the cells were under aerobic conditions, but were kept at 4°C. In some experiments, actinomycin D (Sigma) was substituted for bleomycin.

Bleomycin treatment was expressed in $\mu g\,ml^{-1}$ as is customary through the literature, and MISO in molar concentration. Molecular weight of MISO is 201.2 and thus 2 mM and 5 mM MISO corresponds to 402.4 $\mu g\,ml^{-1}$ and 1006.0 $\mu g\,ml^{-1}$, respectively.

*Hypertonic treatment*

In some experiments, the existence of potentially lethal damage was investigated by treatment of cells with hypertonic solution. In this case, the cells were washed free of the drug under investigation and then they were resuspended in hypertonic solution, 0.5 M NaCl, phosphate buffered saline, pH = 7.2, for 20 min at 37°C. Immediately after hypertonic treatment the cells were resuspended in growth medium and plated.

*Irradiation*

Cells were irradiated under aerobic conditions always prior to MISO toxicity studies. The cells were suspended in growth medium and irradiated at 4°C to the desired dose of X-rays (270 kVp, HVL 1.7 mm Cu, 1.4 Gy $min^{-1}$). Immediately after irradiation, the cells were made hypoxic and MISO treatment started. Thus, no repair time was allowed between irradiation and MISO exposure.

### Results

It has been shown that ionizing radiation reduces the zero-slope shoulder of the cell response to MISO toxicity in hypoxia (Korbelik *et al.*, 1981). An example of this phenomenon is shown in Figure 1a. Cells irradiated to a dose of 7.5 Gy of X-rays, have a shoulder width only 1/3 that of unirradiated

**Figure 1** Radiation-MISO and Bleomycin-MISO treatment (a) CHO cells were irradiated with X-rays to 7.5 Gy (▲) and 11 Gy (▼), and then exposed to MISO for the indicated times under hypoxia. Control cells were not exposed to either bleomycin or radiation (○). (b) CHO cells were exposed to bleomycin, 100 $\mu g\,ml^{-1}$ for 1 h at 37°C. After bleomycin was washed away, the cells were made hypoxic and exposed to 2 mM MISO at 37°C for the indicated times (◇) or they were kept in hypoxia without MISO (★). Immediately after a sample of cell suspension was withdrawn, the cells were washed and plated. The dotted lines are those from panel (a).

cells; a dose of 11 Gy completely eliminated the zero-slope shoulder.

Exposure of cells to bleomycin (100 $\mu g ml^{-1}$, 1 h at 37°C) inactivates CHO cells to the same extent as 11 Gy of X-rays. This treatment also affects the response of surviving cells to MISO toxicity in hypoxia. However, there are important differences compared to the situation with X-rays, as can be seen in Figure 1b. Although the "shoulder" is reduced somewhat by the bleomycin exposure, it is still present; cells inactivated to the same survival level by X-rays completely lost the shoulder. It is clear that there is an initial increase in cell survival under hypoxic incubation with or without MISO present. Beyond 2 h, survival decreases in the presence of 2 mM MISO. The initial increase was absent for aerobic incubation.

The importance of the sequence by which CHO cells are exposed to the drugs is demonstrated in Figure 2. In this case, the cells were always exposed to MISO as follows: 5 mM MISO, 2 h exposure in hypoxia, 37°C. Bleomycin treatment was always for 1 h at 37°C in hypoxia at the indicated bleomycin concentrations. If MISO treatment precedes that of bleomycin, the cell kill was nearly one order of magnitude larger than if the sequence was reversed. Bleomycin is approximately equally cytotoxic to oxic and hypoxic cells. In these studies we limited our experiments to hypoxic conditions.

The possible existence of potentially lethal damage (PLD), inflicted by either bleomycin alone or a bleomycin-MISO combination, was investigated by briefly exposing the cells to hypertonic solution. This procedure has been commonly used to demonstrate the existence of PLD in irradiated cells (Utsumi & Elkind, 1979). It has also been used to demonstrate the existence of PLD in cells exposed to MISO in hypoxia (Korbelik *et al.*, 1982). Figure 3 shows the results of an experiment where cells were first exposed to bleomycin alone (50 $\mu g ml^{-1}$, hypoxia, 1 h 37°C indicated concentrations), or to MISO (5 mM, hypoxia, 2 h 37°C) followed by bleomycin. Immediately after drug exposure, the cells were washed and plated, or they were first treated with a hypertonic solution

**Figure 2** Sequence of bleomycin-MISO treatment. CHO cells were exposed to 5 mM MISO, 2 h at 37°C in hypoxia. This treatment either preceded (▲) or was followed (▼) by exposure of cells to bleomycin (1 h, 37°C in hypoxia). The first drug was washed away prior to the second drug treatment. Control cells were treated with bleomycin alone (○). Immediately after treatment, cells were washed and plated.

**Figure 3** Hypertonic treatment. Cells were treated with bleomycin alone (○, ●) (50 $\mu g ml^{-1}$, 1 h, 37°C in hypoxia) or bleomycin followed by MISO treatment (△, ▲) (5 mM MISO, 2 h at 37°C in hypoxia). Cells were then washed and plated immediately (open symbols) or were first treated with hypertonic solution (0.5 M NaCl, phosphate buffer, pH = 7.2) for 20 min, before plating. The dashed lines are the same data for hypertonic treatment on an expanded scale.

(0.5 M NaCl, phosphate buffer, pH = 7.2) for 20 min at 37°C before plating. The drastic decrease in cell survival after hypertonic treatment suggests the presence of a large amount of PLD-like damage in these cells.

The capacity of cells to repair this damage was investigated and the results of these experiments are presented in Figure 4. Cells were always incubated with 2 mM MISO at 37°C in hypoxia for the indicated times. A sample of cell suspension was withdrawn at the prescribed time, the cells were washed free of MISO and the sample was divided into 3 aliquots. The cells were then exposed to 0, 100 or 300 $\mu g\,ml^{-1}$ of bleomycin, for 1 h at 37°C in hypoxia. Immediately thereafter, bleomycin was washed away and the cells were plated.

**Figure 4** MISO-bleomycin treatment. Cells were exposed to 2 mM MISO at 37°C in hypoxia for the indicated times. They were then washed and further exposed to either 0 $\mu g\,ml^{-1}$ (●), 100 $\mu g\,ml^{-1}$ (△), or 300 $\mu g\,ml^{-1}$ (□) bleomycin for 1 h at 37°C in hypoxia. Immediately after treatment, they were washed and plated. For 2 mM MISO, after 5 h at 37°C in hypoxia, followed by 1 h treatment with 100 $\mu g\,ml^{-1}$ bleomycin at 37°C in hypoxia, cells were plated immediately after washing (△) or, they were further incubated in hypoxia at 37°C for the indicated times, and then plated (▲).

In the case of the 100 $\mu g\,ml^{-1}$ bleomycin exposure, some samples were kept in hypoxia after the last cell wash before they were plated, in order to examine repair of PLD damage. Indeed, keeping cells in hypoxia before plating them resulted in almost a 100-fold increase in cell survival.

### Discussion

The bleomycin-MISO interaction has many similarities to the radiation-MISO interaction. The latter is believed to involve different types of PLD which each modality inflicts on the same target. We believe that this radiation-MISO interaction is simply the result of two types of injuries interfering with each other's respective repair in the same target (Korbelik *et al.,* 1981, and submitted). We would like to put forward a hypothesis that the same mechanism could explain bleomycin-MISO interaction. Bleomycin induces PLD as can be seen from the hypertonic treatment effects, Figure 3. MISO, on its own, has also been shown to induce PLD (Korbelik *et al.,* 1982, and submitted). The combined treatment of cells with both drugs (in sequence) enhances the total PLD (Figure 3).

It was shown that keeping cells in hypoxia after irradiation resulted in increased cell survival (Korbelik *et al.,* and submitted). This was explained as repair of PLD. Cells in hypoxia are arrested in their progression through the cell cycle (Koch *et al.,* 1973), thus giving them time to repair PLD before it is fixed by cell progression. We showed that incubation of cells in hypoxia results in rapid repair of radiation induced PLD, presumably, and there is consequently much less interaction with MISO cytotoxicity (Korbelik *et al.,* and submitted). It is likely that the initial increase in cell survival after bleomycin exposure is also an expression of PLD repair (Figure 1). This is true for cells kept in hypoxia with or without MISO present. The fact that the two curves are indistinguishable up to nearly 3 h, demonstrates that the presence of MISO (2 mM) does not interfere with repair of bleomycin-induced PLD repair. This is consistent with our observation that repair of radiation-induced PLD under hypoxic conditions is not affected by the presence of MISO (Brown *et al.,* 1981; Korbelik *et al.,* and submitted). From the data in Figure 1, it can be estimated that the half time for repair of the bleomycin-induced damage is ~1 h or less. This is almost double the rate found for MISO-induced PLD (Korbelik *et al.,* 1982).

It has been shown for bleomycin MISO combined treatments that the sequences by which the two drugs are used is very important (Roizin-Towle & Hall, 1981 and Figure 2). If bleomycin preceded MISO treatment, there was much less effect on cell killing than when MISO treatment preceded bleomycin. There are at least two possible explanations for this: if the repair kinetics of bleomycin-PLD is much faster that those of MISO-PLD and if repair continues in the presence of the second drug, then this result would be expected. Secondly, if bleomycin PLD is induced more rapidly than MISO PLD, one would again expect this result. It seems that both repair kinetics and damage induction are, in fact, faster for bleomycin. Thus, the importance of the sequence of treatments with the respective drugs can be understood on this basis. Roizin-Towle and Hall (1981) demonstrated that cysteamine plays a protective role in both the MISO pretreatment effect on chemotherapeutic agents and in bleomycin induced cytotoxicity. They suggested that this offers strong evidence of a free radical involvement in the mechanism of enhancement of the action of chemotherapeutic agents by MISO, as well as in the cytotoxicity of bleomycin. In our model, free radicals would have to attack a common target, for example, DNA. Scavengers of free radicals like cysteamine would then protect by depleting the free radicals available to damage the target. It has been demonstrated that MISO treatment of hypoxic cells produces various types of DNA damage in cells (Palcic & Skarsgard, 1978; Wong *et al.*, 1978; Olive, 1979; Taylor *et al.*, 1982; Varghese & Whitmore, 1983). Bleomycin, too, is known to damage DNA and it has been suggested that DNA damage and cell survival are closely correlated (Kohn & Ewig, 1976; Clarkson & Humphrey, 1976; Iqbal *et al.*, 1976; Hurt *et al.*, 1981, 1983; Hurt & Moses, 1984). We thus propose that the common target for cell inactivation with these two drugs is DNA, and that the interaction between the drugs, as measured by cell survival, is in fact the result of interaction of different lesions in the DNA molecule.

In *in vivo* situations, it is possible that these drugs may be much less effective than these *in vitro* results might indicate. In Figure 4, the repair kinetics of combined MISO-bleomycin PLD damage is demonstrated. After treatment, cells were washed free of drugs and were either plated immediately or kept in hypoxia for some time before plating. Further incubation in hypoxia increased the survival nearly 100-fold. If a MISO-bleomycin combination were used in tumour treatments and aimed at chronically hypoxic cells (or even acutely hypoxic cells), then once the drugs were removed by metabolism, repair in hypoxia would greatly diminish their effectiveness. *In vivo,* one would expect hypoxic cells to remain hypoxic for some time after treatment, before being recruited back into the growth compartment, thus they would have time to repair much of the inflicted damage.

It has been demonstrated that hypoxic cells *in vivo* can repair radiation-induced PLD (Urano *et al.,* 1976). This could explain very small additive effects of bleomycin and MISO combination *in vivo* (Stephens *et al.*, 1981; Randhawa *et al.*, 1982).

The MISO-bleomycin interaction reduces the zero-slope shoulder of the MISO toxicity response curve, (Figure 4). This effect resembles that of the radiation-MISO interaction, where it was demonstrated that cells maintained in normal growth conditions for long periods of time could not repair radiation-induced PLD, while in hypoxia the reverse was true (Korbelik *et al.*, and submitted). We have not yet examined the time-course of repair for bleomycin induced PLD under these two conditions.

Other chemotherapeutic drugs may resemble bleomycin with respect to interactions arising from PLD. Preliminary results with actinomycin-D show close parallels to the results reported here for bleomycin.

The authors wish to thank Isabel Harrison and Diane Wurst for their excellent technical assistance. This work was supported by the National Cancer Institute of Canada and the British Columbia Cancer Foundation.

### References

ADAMS, G.E. (1977). Hypoxic cell radiosensitizers for radiotherapy. In: *Cancer: A Comprehensive Treatise.* (Ed. Becker), New York: Plenum Press, Vol. 6. p. 181.

BROWN, J.M., BROWN, D.M., DIONET, C. & HORSMAN, M.R. (1981). Misonidazole inhibits the repair of radiation-induced potentially lethal damage in oxygenated cells. Abstract. *Radiat. Res.*, **87**, 436.

CLARKSON, J.M. & HUMPHREY, R.M. (1976). The significance of DNA damage in the cell cycle sensitivity of Chinese hamster ovary cells to bleomycin. *Cancer Res.*, **36**, 2349.

HALL, E.J. & BIAGLOW, J. (1977). Ro-07-0582 as a radiosensitizer and cytotoxic agent. *Int. J. Radiat. Oncol. Biol. Phys.*, **2**, 521.

HALL, E.J. & ROIZIN-TOWLE, L. (1975). Hypoxic sensitizers: Radiobiological studies at cellular level. *Radiology*, **117**, 453.

HAHN, G.M., RAY, G.R., GORDON, L.F. & KALLMAN, R.R. (1982). Response of solid tumor cells exposed to chemotherapeutic agents *in vivo*: Cell survival after 2 and 24 h exposure. *J. Natl Cancer Inst.*, **50**, 529.

HURT, M.M. & MOSES, R.E. (1984). Abnormal response of xero pigmentosum cells to bleomycin. *Cancer Res.*, **44**, 4396.

HURT, M.M., BEAUDET, A.L. & MOSES, R.E. (1981). Repair of bleomycin-damaged DNA by human fibroblasts. *J. Supramol. Struct. Cell Biochem.*, **16**, 303.

HURT, M.M., BEAUDET, A.L. & MOSES, R.E. (1983). Repair response of human fibroblasts to bleomycin damage. *Mutat. Res.*, **112**, 181.

IQBAL, Z.M., KOHN, K.W., EWIG, R.A.G. & FORNACE, A.J.Jr. (1976). Single-strand scission and repair of DNA in mammalian cells by bleomycin. *Cancer Res.*, **36**, 3834.

KOCH, C.J., KRUUV, J., FREY, H.E. & SNYDER, R.A. (1973). Plateau phase in growth induced hypoxia. *Int. J. Radiat. Biol.*, **23**, 67.

KOHN, K.W. & EWIG, R.A.G. (1976). Effect of pH on the bleomycin-induced DNA single-strand scission in L1210 cells and the relation to cell survival. *Cancer Res.*, **36**, 3839.

KORBELIK, M., PALCIC, B. & SKARSGARD, L.D. (1981). Radiation-enhanced cytotoxicity of misonidazole. *Radiat. Res.*, **88**, 343.

KORBELIK, M., PALCIC, B., SKOV, K. & SKARSGARD, L.D. (1982). Misonidazole and potentially lethal damage. *Int. J. Radiat. Oncol. Biol. Phys.*, **8**, 461.

MOORE, B.A., PALCIC, B. & SKARSGARD, L.D. (1976). Radiosensitizing and toxic effects of the 2-nitroimidazole Ro-07-0582 in hypoxic mammalian cells. *Radiat. Res.*, **67**, 459.

NAKATSUGAWA, S., KADA, T., NIKAIDO, O., TANAKA, Y. & SUGAHARA, T. (1984). PLDR Inhibitors: Their biological and clinical implications. *Br. J. Cancer* (Suppl. VI), **49**, 43.

OLIVE, P.L. (1980). Mechanisms of the *in vitro* toxicity of nitroheterocycles, including flagyl and misonidazole. In: *Radiation Sensitizers: Their Use in the Clinical Management of Cancer.* (Ed. Brady), New York: Mason, p. 39.

PALCIC, B. & SKARSGARD, L.D. (1978). Cytotoxicity of misonidazole and DNA damage in hypoxic mammalian cells. *Br. J. Cancer*, **37**, Suppl. III, 54.

PHILLIPS, T.L., WASSERMAN, T.H., STETZ, J. & BRADY, L.W. (1982). Clinical trials of hypoxic cell sensitizers. *Int. J. Radiol. Oncol. Biol. Phys.*, **8**, 327.

RANDHAWA, V.S., STEWART, F.A. & DENEKAMP, J. (1982). Chemosensitization of mouse tumors by misonidazole. *Int. J. Radiat. Oncol. Biol. Phys.*, **8**, 671.

ROIZIN-TOWLE, L. & HALL, E.J. (1978). Studies with bleomycin and misonidazole on aerated and hypoxic cells. *Br. J. Cancer*, **37**, 254.

ROIZIN-TOWLE, L. & HALL, E.J. (1981). Enhanced cytotoxicity of antineoplastic agents following prolonged exposure to misonidazole. *Br. J. Cancer*, **44**, 201.

STEPHENS, T.C., COURTENAY, V.D., MILLS, J., PEACOCK, J.H., ROSE, C.M. & SPOONER, D. (1981). Enhanced cell killing in Lewis lung carcinoma and a human pancreatic-carcinoma xenograft by the combination of cytotoxic drugs and misonidazole. *Br. J. Cancer*, **43**, 451.

STRATFORD, I.J., ADAMS, G.E., HORSMAN, M.R. & 4 others. (1980). The interaction of misonidazole with radiation, chemotherapeutic agents, or heat: A preliminary report. *Cancer Clin. Trials*, **3**, 231.

TAYLOR, Y.C., BUMP, E.A. & BROWN, J.M. (1982). Studies on the mechanism of chemosensitization by misonidazole *in vitro*. *Int. J. Radiat. Oncol. Biol. Phys.*, **8**, 705.

URANO, NESUMI, N., ANDO, K., KOIKE, S. & OHNUMA, N. (1976). Repair of potentially lethal radiation damage in acute and chronically hypoxic tumor cells *in vivo*. *Radiology*, **118**, 447.

UTSUMI, H. & ELKIND, M.M. (1979). Potentially lethal damage versus sub-lethal damage: Independent repair processes in actively growing Chinese hamster cells. *Radiat. Res.*, **77**, 346.

VARGHESE, A.J. & WHITMORE, G.F. (1984). Detection of reactive metabolite of misonidazole in hypoxic mammalian cells. *Radiat. Res.*, **97**, 262.

WONG, T.W., WHITMORE, G.F. & GULYAS, S. (1978). Studies on the toxicity and radiosensitizing ability of misonidazole under conditions of prolonged incubation. *Radiat. Res.*, **75**, 541.